# Mental time travel ability influences the representation of events and emotional expressions: evidence from microblogs

**DOI:** 10.1186/s40359-023-01096-4

**Published:** 2023-03-03

**Authors:** Zaoyi Sun, Qingyan Li, Fei Luo, Liang Xu

**Affiliations:** College of Education, Zhejiang University of Technology, Hangzhou, 310023 China

**Keywords:** Mental time travel, Microblog, Text representation, Emotional expressions

## Abstract

Mental time travel (MTT) ability allows people to project themselves mentally into the past and future. It is associated with people’s mental representation of events and objects. Using text analysis methods, we explore the linguistic representation and emotional expression of people with various MTT abilities. In Study 1, we assessed the users’ MTT distances, text lengths, visual perspectives, priming effects of temporal words, and emotional valences by analyzing 2973 users’ microblog texts. From our statistical analysis findings, users with far MTT incorporated longer text length and more third-person pronouns in their microblogs and are more likely to relate the future and past with the present than people with near MTT. However, the study showed no significant difference in emotional valence between people with different MTT distances. In Study 2, we explored the relationship between emotional valence and MTT ability by analyzing the comments of 1112 users on “procrastination.” We found the users with far MTT more positive toward procrastination than those with near MTT. By analyzing users’ social media platform data, this study re-examined and verified previous findings indicating that users who mentally travel different temporal distances represent events and emotional expressions differently. This study serves as an important reference for MTT studies.

## Background

It is often said that “only by remembering the past can we look forward to the future.” People can remember their past and plan for the future [1, 2]. The human ability to remember and reconstruct past events and visualize and anticipate future events is defined as mental time travel (MTT) [3–5]. This is through a human’s episodic memory as the events in mental time are constructed around personally experienced past and future constructs [6]. Specifically, the episodic memory’s main role is to provide information from the past as the scaffolding of the future. Various lines of evidence suggest that MTT into the past shares cognitive resources with mental construction of potential future episodes. For example, participants reported a decrease in phenomenological richness of both past and future episodes with increased distance from the present[7]. Empirical studies have found that the temporal distribution of past events people envisage follows the same power function as the temporal distribution of anticipated future events [8]. MTT has a considerable association with an individual’s cognition, emotion, and behaviors. Extensive researches has showed that mentally traveling into the past allows people to review events, draw lessons from previous experiences, and respond to present and future issues better [9], whereas mentally traveling into the future may help people adjust their current actions by pre-experiencing future events [10]. A prior study found that children between the ages of three to five began to develop MTT ability so that their current behavior could be directed to secure not just present, but individually anticipated future needs [11]. Therefore, MTT allows people to adapt to the past and present and combine existing experiences to make better plans.

MTT is a fundamental characteristic of people mentally representing events and objects [12]. Construal level theory (CLT) suggests that people’s mental representation of events and objects depends on the temporal distance at which those events and objects are imagined [13, 14]. Recently, a series of experiments have found that thinking about events that are close in time induces a “low-level construal”, characterized by more concrete or detailed processing related to the event. In contrast, thinking about events that are farther away induces a “high-level construal,” characterized by more abstract or holistic processing[15]. CLT considers the temporal distance effects and focuses on the events and objects, whereas MTT focuses on the mental ability of individuals. The ability to travel mentally at different temporal distances may affect the individuals’ representation of events and behavioral patterns [16, 17]. The increased mental temporal distance made people integrate more information and improve their comprehensive decision making [18]. People with near-future sightedness are more likely to be addicted to alcohol and gambling [19], and people with far-future sightedness behave more cautiously in long-term savings [20].

The concept of time perspective emerged in psychology as a way to understand how people cognize, understand, and feel time [21]. Time perspective is related to many outcomes of interests such as well-being and achievement, which explains the propensity of individuals for MTT [22]. Although the time perspective includes three basic elements (the perception and experience of past, present, and future), many studies only investigated the influences of one temporal perspective on human behavior [23, 24]. Recent studies have shown that the regions of the brain used for thinking about the past are also active during the prospective thinking process [25–27]. In addition, many studies have used the questionnaire method or lab experiments to investigate the relationship between people’s MTT abilities and representation of events, limiting them in terms of sample size and ecological validity. Considering the accessibility of online big data, this study proposes to mine users’ text data on social media platforms and investigate how people’s MTT abilities affect their text representation and emotional expression.

### MTT and text representation

Studies have been increasingly exploring the relationship between temporal distance and event representation in recent years. Some natural studies based on CLT have found that people’s representation of far-future events contains more goal-related knowledge, and representation of near-future events containing more details of actions [28]. Some researchers investigated the relationship between temporal distances and the level of lexical abstractness by analyzing the word meanings in a large language corpus [29]. They found that the tweets mentioning temporally proximate dates used more concrete words than those mentioning distant dates. Based on the data extracted from the New York Times Annotated Corpus, a previous study conducted factor analysis and generated a “Conceptual Abstractness and Uncertainty” dimension underlying the co-occurring patterns [30]. The results confirmed a positive correlation between temporal distance and conceptual abstractness/uncertainty. The present study also proposes to investigate how people’s perception of time affected their representations in social media text. However, quantifying the representation of abstract concepts remains a challenge. The above studies relied on manual evaluation under different rules, but this method has limitations in terms of efficiency and objectivity. Following previous quantitative linguistic studies [31], we considered text length as a more objective and measurable variable for automatic computing text length is one of the main indexes of complexity [32]. Besides, a related work accessed text’s abstractness by the frequency of concrete nouns and the average abstractness of nouns [33]. For further analysis of abstractedness in our study, a dictionary of abstractness ratings for 9877 two-character Chinese words was used for lexical matching [34]. So we defined “abstractedness” with these indices: the average abstractness of texts, the frequency of abstract words and the text length. Therefore, we assume that people with far MTT prefer to use longer text, more abstract words and higher abstractness ratings of texts on social media platforms compared to those with near MTT (Hypothesis 1).

A visual perspective is the use of different personal pronouns to describe past or imagined events[35], this is an important factor in MTT research as well. Experimental and natural studies have found that people tend to use a third-person rather than first-person perspective for remembering distant past events [36–40]. The visual perspective is related with individual’s two cognition process defined by the origin of information: top-down and bottom-up. Top-down signals were derived from the knowledge about the task volitionally whereas “bottom-up” signals were derived from salient stimuli automatically[41]. Third-person perspective is more appropriate for “top-down” abstractions and the first-person perspective more appropriate for concrete and “bottom-up” processing [42]. Thus, the longer the time distance, the more people are inclined to abstract “top-down” cognitive processing. People mentally traveling to the future tended to use more third-person pronouns as the temporal distance increased [27]. This study proposes to retest the above findings by analyzing users’ social media text. We hypothesize that people with far MTT use more third-person pronouns in their blogs than people with near MTT (Hypothesis 2).

In the field of semantic priming, the priming effect was first defined as facilitating the recognition process by spreading activation from one word to neighboring words [43]. Individuals form these activations to link concepts in mind. These activation tags are more accessible in one’s mind and affect the evaluation of subsequent information [44]. For example, people tended to recognize the word school faster when it was preceded by the word teacher than when it was preceded by the word bread. In the case of temporal orientation, some researchers redefined the priming effect as the increased probability of referring to the future and past after referring to the present [23]. By identifying tweets that referred to the present, they examined the tweets that followed within 3 min of that tweet were examined for the proportions of times that they mentioned the past, present, and future. These likelihoods were compared with the likelihood of past, present, and future references in tweets not occurring within 3 min of a present reference. Based on the user’s natural language on social platform, this work adopted the text analysis methods to verify the references to the present primed references to the future, and such priming effects was stronger for the people with far future temporal distance. These results can be explained by CLT [13, 14] that people who look far into the future tend to talk less concretely about the present. Furthermore, the point where the level of abstractness in the present matches the level of abstractness in the future will tend to be farther into the future (and past) for those with far MTT than for those with MTT. This method inspired us to explore whether the users of Chinese social platforms with far (vs. near) MTT tend to relate the present more (vs. less) with the future and past. Following the previous work, we focus on the users’ MTT in both past and future directions. We thus hypothesize that people with far MTT have stronger priming effects; that is, they tend to mention more past and future references after the present references(Hypothesis 3).

### MTT and emotional expressions

Studies have found that the perceived valence (positive or negative) of events is influenced by its temporal distance. Participants were asked to provide an important event that they had experienced in the past or would experience in the future, and indicate how positive the emotions for the event they experienced. It was found that distant past events were generally more negative than recent ones; in contrast, far future events were more positive and higher in their relation to identity and their effect on the self [45]. The above studies focus on how the emotional valences of events changed with different temporal distances from past and future time orientations. This study proposes to explore how peoples’ MTT abilities influence their emotional expressions. As an enhanced activation, the neural mechanism of future MTT has been found to occur in areas of the brain related to optimism [46]. Since MTT is an integrated time perception mechanism, we hypothesize that the text expressions in emotional valence are different between people with far and near MTT abilities(Hypothesis 4).

### The present study

In sum, this study investigated the text representation and emotional expression of people with different MTT abilities by analyzing their microblogs. In Study 1, we calculated the users’ MTT, text length, words’ abstractness, visual perspectives, priming effects of temporal words, and expressions of emotional valences. We hypothesized that people with far MTT present longer text length, higher abstractness ratings of words, use more third-person pronouns, show stronger priming effects, and reveal more positive emotional valence in their text than those with near MTT. In Study 2, we further explored the relationship between people’s emotional valence and MTT ability by analyzing their text under the topic “procrastination.”

## Study 1

In Study 1, we analyzed text based on the original content on Sina Weibo. Sina Weibo, or microblogs, a Chinese version of Twitter, is the largest social media platform in China [47]. According the report released by China Internet Network Information Center, 55.3% of Chinese netizens use Sina Weibo as their major social media software. Based on user relationships, Weibo realize information sharing, dissemination and acquisition. Therefore, with the representative samples and rich user-generated content, Sina Weibo enabled us to the text’s characteristics of people with different MTT abilities.

### Method

#### Data collection procedure

This study did not require an ethics approval, as it was a text mining retrospective study of publicly posted information on social media. Following the principle of intensity sampling [48], for each province of China, the users who had at least 100 original microblogs were selected as they could more information for the text analysis. We downloaded 236,629 original microblogs of 3850 users who had at least 100 original microblogs each. We collated the users’ personal information, such as gender and number of followers. As users with numerous followers are usually affiliated with professional organizations, we deleted those with more than 10,000 followers because most of their microblogs contain advertisements, which may have distorted the results. Our final sample included 213,201 microblogs of 2973 users (sample utilization rate: 77.42%; 81.32% females). The regional distribution of the simple covered 34 provinces and the top three were Guizhou Province (4.5%), Sichuan Province (4.1%) and Zhejiang Province (4.0%).

#### Feature calculation instruments

##### MTT ability

The temporal reference words appearing in microblog text can help in quantifying users’ MTT distance [23]. For example, in the sentence “it will rain tomorrow,” the word “tomorrow” is a future indicator. The Chinese language, unlike English, has no specific grammatical rules to distinguish the different directions of time. Therefore, we first constructed a corpus of temporal reference words for past, present, and future directions based on a Chinese temporal corpus [49]. A total of 287 words and rules were contained in the corpus. Second, we converted the above Chinese temporal references into numerical expressions by the rules of SUTime [50]. SUTime is a rule-based temporal tagger for recognizing and normalizing temporal expressions in English text. It supports three basic types of time objects: Time, Duration, Interval. A time point indicating a particular instance on a time scale. SUTime recognize both relative times (e.g., last Friday) and absolute times (e.g., December 11, 2022). Duration refers to a combination of a unit (e.g., day, month, year, etc.) and a numeric value (the quantity associated with the unit). Interval means a time range defined by a start and end time points, such as November 12 to December 10, 2022. SUTime can recognize these ranges and represent them a Duration with begin and end times. Thus, the Chinese temporal reference words we built were classified into these types and then the user’s MTT distance could be recognized automatically. The above temporal references and rules together allowed SUTime to recognize the time-related expressions in microblog text automatically. Third, we extracted users’ MTT distances from the microblogs using the SUTime temporal tagger. The microblogs were classified under absolute dates, relative time, and a mixture of absolute and relative time. For relative time, we used document dates as references, while SUTime estimated the MTT distance of a microblog as follows: the time at which the microblog was created subtracted from the date and time referred to by the microblog. As there is more than one temporal reference in a microblog, the MTT distance is the average of these references; that is, ${\text{S}}\left( {\text{t}} \right) = \sum\nolimits_{{\text{r}}} {\frac{{{\text{M}}\left( {\text{r}} \right)}}{{\text{n}}}}$, where *r* is the temporal reference, *M*(*r*) is the number of days the reference projects into the past or future, and *n* is the number of temporal references in the microblog. User *i*’s MTT microblog distance is then averaged as follows: ${\text{S}}\left( {\text{i}} \right) = \sum\nolimits_{{\text{t}}} {\frac{{{\text{S}}\left( {\text{t}} \right)}}{{\text{n}}}}$, where *t* is one of the user’s microblogs and *n* is the total number of microblogs created by user *i*.

##### Measurement of abstractedness

We quantified "abstractedness" with these indices: the average abstractness of a single microblog, the average abstractness of a single word and the text length. The text length of one’s microblog is the average length of the microblog, indexed as the microblog’s total number of words divided by the number of microblogs as follows:

${\text{L}}\left( {\text{i}} \right) = \sum\nolimits_{{\text{t}}} {\frac{{{\text{L}}\left( {\text{t}} \right)}}{{\text{n}}}}$,where *t* is the user’s microblog, *L*(*t*) is the total number of words in the microblog, and *n* is the user’s number of microblogs.

The average abstractness of words was indexed as the total words' abstractedness ratings divided by the number of user's microblogs. The average abstractness of a single word was indexed as the frequency of abstract words in the abstractness dictionary divided by the total number of words in the user’s original microblogs.

##### Visual perspective

As an objective measurement of language vocabulary, language inquiry and vocabulary counting (LIWC) is a computerized text analysis program for classifying and quantifying the user’s features of language application [51]. To apply this method to simplified Chinese, a simplified Chinese LIWC (SC-LIWC) program was established based on the original LIWC [52]. SC-LIWC contains the categories of first and third person pronouns useful for our visual perspective analysis. The words used in the microblogs are then matched with the above personal pronouns. Finally, the user’s first/third-person perspective can be calculated as follows:

$$\it {\text{F}}\left( {\text{i}} \right) = \sum\nolimits_{{\text{t}}} {\frac{{{\text{F}}\left( {\text{t}} \right)}}{{\text{n}}}}$$ ,

where *t* is user *i*’s microblog, *F* (*t*) is the frequency of using first/third-person pronouns in the microblog, and *n* is the user’s total number of microblogs.

##### Priming effects of temporal words

We used a temporal reference corpus for the MTT distance calculation processing. Each user’s temporal references are identified using SUTime. We then used the proportions of past and future references to compute the priming condition (in microblogs occurring 0–3 h after a present reference) and non-priming condition (in all other microblogs).

The index of priming effects is the proportion of past and future references occurring 0–3 h after a present reference is divided by the proportion of the references not occurring after the present reference.

##### Emotional valence

Emotional valence is evaluated using a simplified Chinese version of the National Taiwan University Sentiment Dictionary (NTUSD), which includes 2810 positive and 8276 negative words [53]. The NTUSD automatically labeled the user’s emotional words found in the microblogs during the lexical matching process. For each user, one positive word is given 1 point and one negative word is given − 1 point. The average emotional valence score of user *t* is then indexed as the sum of the positive and negative word scores as follows:

$${\text{C}}\left( {\text{t}} \right) = \frac{{\sum\nolimits_{{\text{p}}} {{\text{E}}\left( {\text{p}} \right)} + \sum\nolimits_{{\text{n}}} {{\text{E}}\left( {\text{n}} \right)} }}{{\text{N}}},$$

where *p* is a positive emotional word, *n* is a negative emotional word, *E* (*p*) is the total score of user *t*’s positive emotional words, *E* (*n*) is the total score of user *t*’s negative emotional words, and *N* is the total number of user *t*’s emotional words (including positive and negative words).

#### Data analysis

All analyses were conducted using IBM SPSS v.23. The descriptive statistics of the variables are shown in Table 1. Then we first used the Kolmogorov–Smirnov test (*KS* test) to examine the normality of sample distribution. We found that the MTT distance (*KS* = 0.45), text length (*KS* = 0.19), the average abstractness of a single word (*KS* = 0.07), the average abstractness of a single microblog (*KS* = 0.12) and emotional valence (*KS* = 0.15) are non-normally distributed (all *p* < 0.001). Therefore, the Mann–Whitney U-test was conducted for the future different test. *r* can be calculated as an effect size for the Mann–Whitney U-test using the formula: $r=\frac{z}{\sqrt{N}}$ [54]. According to Cohen’s guidelines, a large effect is 0.5, a medium effect is 0.3, and a small effect is 0.1 [55].

**Table 1:** Descriptive statistics of variables in study 1

	*Min*	*Max*	*M*	*SD*
Mental time travel distance	0.00	8396.74	44.44	357.17
Emotional valence score	− 1.00	1.00	0.24	0.51
Text length	0.10	99.03	10.40	11.87
The average abstractness of a single word	0.02	0.32	0.13	0.02
The average abstractness of a single microblog	0.00	0.91	0.34	0.08

Second, a Spearman correlation analysis showed that the user’s MTT distance is positively correlated with the text length (*r* = 0.61, *p* < 0.001), the average abstractness of a single microblog (*r* = 0.06, *p* < 0.05) and the average abstractness of a single word (*r* = 0.08, *p* < 0.01). The text length is positively correlated with the average abstractness of a single microblog (*r* = 0.12, *p* < 0.001) and the average abstractness of a single word (*r* = 0.14, *p* < 0.001). The correlations between the other variables are not significant (*p* > 0.05).

According to the critical ratio method, the MTT distances of all the users were sorted, the first 27% were enrolled in the far MTT group (73.5% females), and the last 27% were enrolled in the near group (84.4% females) for the further analysis.

### Results

#### Indices of abstractedness

We then conducted a Mann–Whitney U test to examine the differences in the average abstractness of a single microblog, the average abstractness of a single word and the text length between the near MTT and far MTT groups. As shown in Table 2, all the three variables of users with far MTT was significantly higher than that of users with near MTT *p* < 0.001), supporting Hypothesis 1.

**Table 2:** The differences in the average abstractness of a single word, the average abstractness of a single microblog and the text length between the near MTT and far MTT groups

	The average abstractness of a single word	The average abstractness of a single microblog	Text length
Far MTT	830.75	830.46	1098.67
Near MTT	758.34	759.68	508.33
Z score	3.15**	3.07***	25.51***
Effect size *r*	0.08	0.08	0.64

**p < 0.01; ***p < 0.001

#### Visual perspectives

**Fig. 1:**
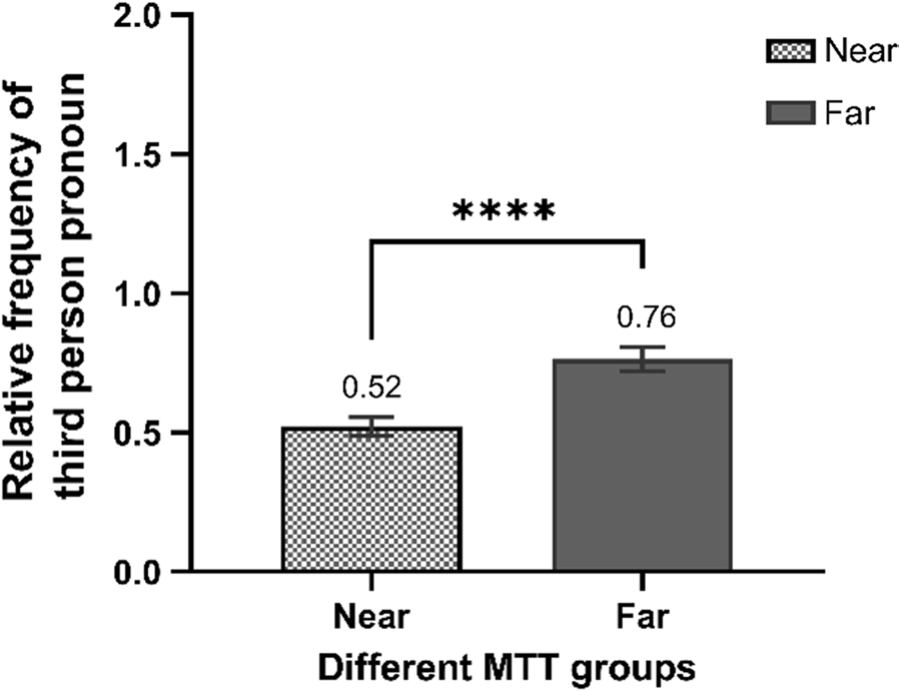
The difference in relative frequency of personal perspective between near versus far MTT groups. Error bars: the relative frequency of personal perspective’s standard error (*SE*) of each group. ***: significant at 0.001 level.”

As the data were non-normally distributed (first-person pronoun frequency *KS* = 0.34, third-person pronoun frequency *KS* = 0.49; all *p* < 0.001), we performed a Mann–Whitney *U* test to compare the usage of personal pronouns between the far MTT and near MTT groups. As shown in Fig. 1, the results indicated a significant difference in frequency of third-person pronoun use between the far MTT and near MTT groups (Z_0.01/2_ = 4.33, *p* < 0.001, effect size: *r* = 0.12). Individuals who travel farther in mental time are more likely to use third-person pronouns.

#### Priming effects analysis

We calculated the frequencies of future and past temporal word use of those with different MTT distances under both priming and non-priming conditions. As the data did not conform to a normal distribution (all *p* < 0.001), we used the Mann–Whitney U test for the difference test. As shown in Table 3, there were significant differences between the near MTT and far MTT groups in both future and past temporal word usage under priming and non-priming conditions (all *p* < 0.001). Specifically, users with far MTT are more likely to use past and future temporal words in both conditions. Thus, compared with users with near MTT, those with far MTT are more likely to prime by the present references and regard the future and past more associated with the present.

**Table 3:** The differences of temporal words’ frequencies mentioned by users of different MTT distances under priming and non-priming conditions

	Priming		Non-priming	
	Past temporal words frequency	Future temporal words frequency	Past temporal words frequency	Future temporal words frequency
Far MTT	1038.53	990.24	845.18	834.83
Near MTT	568.47	616.76	761.82	772.17
Z score	24.34***	20.81***	8.72***	6.94***
Effect size* r*	0.61	0.52	0.22	0.17

***p < .001

As a user may refer to many temporal words in one priming procedure, we calculated the users’ frequency of future (or past) temporal word usage divided by the total prime time as the primed temporal words’ strength. We also calculated the strength of the non-primed temporal words using this method. We then conducted a Mann–Whitney U test to examine the difference between the two MTT groups in the strength of the above temporal words. The strengths were consistent (see Table 4) with the frequencies (see Table 3). The far MTT group used more past and future temporal words after mentioning the present temporal words in one priming procedure than did the near MTT group (all *p* < 0.001). From these results, far MTT users may have a stronger priming effect, thus supporting Hypothesis 3.

**Table 4:** The differences of temporal words’ strengths mentioned by users of different MTT distances under priming and non-priming conditions

	Priming			Non-priming		
	Past words strength	Future words strength	Strength difference	Past words strength	Future words strength	Strength difference
Far MTT distance	938.92	844.80	903.70	921.66	834.18	894.43
Near MTT distance	668.08	762.20	703.30	685.34	772.82	712.57
Z score	17.55***	8.64***	11.92***	16.03***	6.80***	11.24***
Effect size* r*	0.44	0.22	0.30	0.40	0.17	0.28

***p < .001

#### Emotional valence

**Fig. 2:**
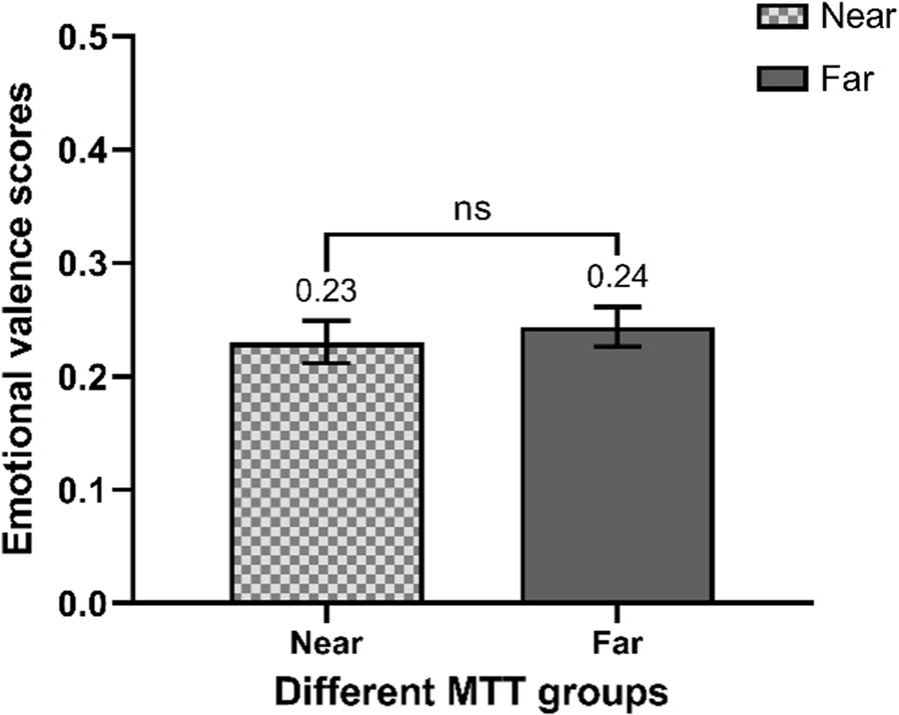
Emotional valence scores of near and far MTT distance groups. Error bars: the relative frequency of personal perspective’s standard error (*SE*) of each group. ***: significant at 0.001 level.”

After calculating the users’ emotional valence scores (*M* = 0.23, *SD* = 0.53), we conducted a Mann–Whitney U test to analyze the difference in emotional valence between the far MTT and near MTT groups. The results showed no significant difference in emotional valence scores between the two groups (see Fig. 2: *Z*_0.05/2_ = − 0.99,* p* = 0.3 *r* = − 0.02), not supporting Hypothesis 4.

### Discussion

Study 1 investigated the difference between the far MTT and near MTT groups in text representation and emotional expression by analyzing the users’ microblogs. We found that far MTT users have more abstract expressions, stronger priming effects, and more third-person pronoun use in their original content than near MTT users.

Previous studies have found that the temporal distance of an event positively correlated with its representational abstractness [29]. Using the text analysis method, we found the users’ MTT positively correlated with the text length, the average abstractness of a single microblog and the average abstractness of a single word. However, the effect sizes of the abstractness-related indexes were small when the number of words or microblogs were controlled. These results indicated that the people with far MTT tended to represent events with longer text, not just more abstract words. Computational linguistics studies have often adopted text length as an index of content complexity to find that language complexity is positively correlated with an event’s temporal distance [56]. Therefore, our findings may indicate that people with far MTT show higher language complexity. In addition, some studies have found that low-level future time perspective individuals describe their future events vividly [57]. These findings agree with our priming effects results that people with far MTT tend to use more temporal words (past and future) in both priming and non-priming conditions. Thus, individuals with far MTT refer to both events with far temporal distance and temporal related events more frequently.

We also found that users with far MTT tended to use more third-person pronouns in their original text. This finding agrees with previous research that as the temporal distance increased, the user tended to adopt the observer perspective more frequently [27]. This difference in expression style reflects the user’s cognitive tendency at different temporal distances. Specifically, with the increase in time distance, the user’s involvement degree decreased, and the user tended to adopt observer perspectives more frequently as the event was formed with less clarity and intensity [3].

For the priming effect, we found results similar to the findings that users with far MTT referred to more temporal words after mentioning present events than did those with near MTT [23]. The priming effect results indicated that MTT allows users to connect the past and future with the present. This is consistent with the definition of MTT that people use this ability to reflect on the past, anticipate the future, and construct alternate mental realities [58].

Generally, Study 1 found that people’s MTT affects their language representation, including their text length, abstractness of words, visual perspective usage, and priming effects of temporal words. These results can be explained by the CLT theory that temporal distance influences individuals’ cognitive style toward events. However, we found no significant difference between the far MTT and near MTT groups in terms of emotional valence. One possible reason is that a user’s microblog may involve various event types and emotional valences. An analysis of the whole content cannot distinguish the group differences toward a specific event because event type is an important factor affecting the relationship between emotional expression and temporal distance [7]. Therefore, in Study 2, we further analyzed the differences in emotional valence toward a specific topic between the far MTT and near MTT groups. As Study 1 integrated the future and past time orientations as the overall MTT ability of each user, in this study, we distinguished between the two time orientations.

## Study 2

Study 1 indicated that the overall emotional valences of users with different MTT distances were not significantly different. As the user’s original content consisted of different types of topics, the results could be misleading. Therefore, Study 2 selected the topic of procrastination for further analysis. Procrastination implies the lack of self-regulation and a behavioral tendency to postpone what is necessary for goal achievement [59]. Procrastination is always a self-handicapping and dysfunctional behavior [60]. The reasons we focused on the posts about procrastination are as follows: First, in order avoid the floor effect and ceiling effect, the topic selected in study 2 should not evoke most people’s congruent emotional valence. According to the related works, people expressed diversified attitudes towards the topic of “procrastination”. Second, Time management is a strong factor in mediating between procrastination and self-regulation [61]. As referred in the Introduction, People’s MTT ability has important effect on time management behavior. Previous studies focused on the relationship between future temporal perspective (i.e. goal-setting) and procrastination [61]. Therefore, the users in study 2 were divided into 4 groups in order to clarify the relationship between MTT abilities and emotional valences toward procrastination for both past and future temporal orientations.

Thus, we selected this topic in study 2 to explore the relationship between user’s MTT ability and emotional valences towards procrastination. Third, as a widespread problematic behavior and a stable individual tendency across time and different contexts, procrastination is familiar to most people in China over recent years. Many users posted their attitudes, feelings, experiences under this topic, which offered nature and rich resources for our research. In this study, we considered the user’s content on procrastination and compared the emotional valences of the far MTT and near MTT groups. We also hope that these results may provide an insight into how people’s MTT abilities affect their attitudes towards procrastination, which would have potential implications for effective prevention of procrastination.

### Method

#### Data collection procedure

This study did not require an ethics approval, as it was a retrospective text mining study of publicly posted information on social media. We downloaded 4605 comments of microblog users on procrastination and collected the users’ original microblog texts using their user IDs and their comments. As in Study 1, we first removed the data of users who had more than 800 followers to finally obtain 1864 users with a total of 145,010 microblogs. Second, we filtered the data of 625 users who had no temporal or emotional words in their original content. Third, we merged the data of users having the same ID. Finally, we included the data of 1112 users for the following analysis (83% women).

#### Feature calculation instruments

As in Study 1, we used a simplified Chinese version of the NTUSD and a self-constructed temporal word corpus in Study 2. We also calculated each user’s average MTT distance and emotional valence in the same manner as in Study 1.

#### 3.1.3 Data analysis

The user’s emotional valence scores showed a mean value of − 0.02 (*SD* = 0.07). As the distribution of all the users’ emotional valence scores and MTT distances was non-normal (*p* < 0.001), we conducted a Spearman rank correlation analysis. As shown in Table 5, the users’ average emotional valence scores were positively correlated with the past and future MTT distances (both *p* < 0.001). From these results, the farther a user’s MTT, the more positive is their attitude toward procrastination. We also found a positive correlation between the users’ past and future mental travel distances (*p* < 0.001), suggesting that the users’ past and future mental distances were intrinsically connected.

**Table 5:** Spearman rank correlation analysis of emotional valence and MTT distances

	Past MTT distance	Future MTT distance	Emotional valence
Past MTT distance	*r* = 1.00		
Future MTT distance	*r* = 0.26***	*r* = 1.00	
Emotional valence	*r* = 0.32***	*r* = 0.25***	*r* = 1.00

***p < .001

### Results

#### Difference test in emotional valence

The users’ data were grouped by the past and future MTT distance mean scores and labeled under four groups: far-past MTT (*n* = 235), near-past MTT (*n* = 877), far-future MTT (*n* = 184), and near-future MTT (*n* = 928). Notably, a user may belong to more than one group. As the past and future MTT abilities are intrinsically connected, users with far-past MTT ability are likely to also have far-future MTT ability. Because the users’ MTT distance distribution is non-normal, we used a Mann–Whitney U test for the difference test. For both past and future temporal orientations, we found that the far MTT group tended to describe procrastination more positively than the near MTT group (past: *T*_past−far_ = 643.28 > *T*_past−near_ = 533.25, *Z*_0.01/2_ = 4.67, *p* < 0.001, effect size *r* = 0.12); future: *T*_future−far_ = 645.68 > *T*_future−near_ = 538.82, *Z*_0.01/2_ = 4.13, *p* < 0.001, effect size *r* = 0.10).

### Discussion

In Study 2, we analyzed the users’ comments under procrastination, exploring whether those with different MTT distances showed different emotional valence. We found that in both past and future temporal orientations, the far MTT group described procrastination more positively than the near MTT group. These findings support Hypothesis 4.

The results of study 2 suggest that the user’s time perception is related to the attitude towards procrastination. Procrastination is a dysfunctional behavior related to failing in meeting deadlines within a specific time-frame. Although people generally consider procrastination a stressful event in daily life, all procrastination behaviors do not have negative effects. Some researchers conceptually differentiated between two types of procrastinators: passive procrastinators and active procrastinators [62]. Passive procrastinators do not procrastinate intentionally but always find it difficult to make decisions. They then act quickly to complete their tasks on time. In contrast, active procrastinators handle their issues in a timely manner, suspend their issues deliberately, and pay attention to other valuable work at hand [63]. Passive and active procrastinators have different cognitive, affective, and behavioral dimensions [64]. Active procrastination reflects good self-regulation and planning [65]. Self-regulation includes self-generated thoughts, feelings, and actions that are directed towards the attainment of personal goals [66]. Procrastination has been increasingly viewed as involving a failure in self-regulation [67]. Specifically, passive and active procrastinators have differences in the elasticity in time perception and use patterns. Positive procrastinators are able to properly estimate the minimum amount of time required to finish a task and push themselves to proceed efficiently toward the future goal, whereas passive procrastinators tend to underestimate the time needed to complete a particular task and thus to be overwhelmed or overstressed by time pressure at the last minute [62]. Therefore, our results were consistent with previous works that time management is a strong factor in mediating between procrastination and self-regulation [68].

Procrastination may have different meanings for users with different MTT abilities because a person with far MTT usually adopts more long-term planning [20]. Our results indicate that people with different MTTs represented different emotional valences toward procrastination. Users with far MTT regard procrastination more positively. A possible explanation is that people with far MTT may engage in active procrastination and strategically adopt decisions to complete their tasks before the deadline methodically. Far MTT ability allows people with “foresight” to establish a comprehensive vision and reasonably arrange and allocate what they want to do [65]. Therefore, people with far MTT ability focus on the task at hand when adopting active procrastination.

We also found a positive correlation between past and future MTT distances (*p* < 0.001), indicating that people with far MTT would travel farther in both past and future orientations. In addition, Study 2 found that regardless of past and future orientations, people with far MTT are more positive toward procrastination. In Study 1, the priming effects of past and future temporal words showed similar tendencies, indicating that MTT may have a common mechanism in the two orientations. The results of Study 2 indicated it’s reasonable and feasible to comprehensively consider the two temporal orientations of MTT as a whole ability of time perception.

## General discussion

From the perspective of social psychology, people’s MTT ability is highly correlated with their social cognitive ability [58]. The language people use on social platforms reflects what they are thinking about and how they organize and analyze the environment [69]. Unlike self-report surveys and lab experiments, language is a natural and reliable mode for people to translate their thoughts into records that others can understand [70]. Psychologists have recently explored people’s cognitive abilities through the text analysis of user-generated content (UGC) [71].

Unlike previous studies that mainly adopted surveys and lab experiments to explain the cognitive characteristics of a person’s MTT ability, we explored the difference in text representation and emotional expression between users with different MTT abilities through text analysis. The findings of Study 1 support the conclusions of CLT and other related work that temporal distance influences people’s cognition and behavior patterns. Furthermore, Study 2 showed that users with different MTT distances have different emotional valances toward procrastination. This finding is intriguing because it implies that how people cognitively engage with time influences how they cognitively engage with social events [72]. This means that, for the same event, people with different MTT abilities would adopt different cognition styles and express different emotions.

In contrast to previous studies that focused on one orientation of the time perspective, we explored the users’ MTT in both past and future orientations. Consistent with previous findings that an individual’s past and future MTT shared some common neural networks and cognitive structures [73], we found that the users’ past and future MTT distances positively correlated. People’s past and future MTT orientations are influenced by their inner instructions in a similar way and generate external versus internal events as well as positive versus negative events at different temporal distances from the present [26]. Brain imaging studies have shown a great amount of overlapping neural activity while visualizing personal past versus future experiences [74]. Therefore, our findings support the above conclusions.

Finally, this study has several limitations as well as future directions. First, the gender ratio of the sample data was unbalanced, with a higher proportion of female than male users (approximately: Study 1—men: women = 1:4; Study 2—men: women = 1:5). In fact, women are more likely to use social networking sites [75] and express opinions and emotions in online social platforms than males [76].Therefore, future studies could explore the MTT effect by gender. Second, as age is sensitive and private information for some people, some users’ age information was not provided or randomly given (e.g., 120 years old). Thus, we could not analyze the users’ MTT distances by age group. Future studies could collect data with more valid age information and explore the MTT abilities by age group. Although Weibo is the largest social media platform in China, the present findings should be further examined considering the possible differences in some personality traits between Weibo users and non-users. Third, we constructed a Chinese temporal lexicon based on an English temporal lexicon. However, owing to semantic fuzziness in the Chinese language, we could not recognize all the temporal expressions. Therefore, future studies could optimize the Chinese temporal lexicon used in this study by using advanced text mining algorithms.

## Conclusions

This study explored the text representation and emotional expression of users with different MTT distances by analyzing their original texts in Sina microblogs. Study 1 found the users’ text representation related to their MTT abilities through text analysis. Specifically, compared to users with near MTT, those with far MTT provided longer text length, were more involved in observer perspectives, and had a stronger priming effect of temporal words. As for emotional expression, the whole text showed no significant difference between users with far and near MTTs. Study 2 examined the relationship between users’ emotional valences toward procrastination and their MTT abilities. It found that users with far MTT were more positive toward procrastination than those with near MTT.

## Data Availability

The data can be made available upon reasonable request from the corresponding author.
